# Retinal mitochondrial Fluorescence‐Lifetime Ophthalmoscopy data in patients with Alzheimer’s Disease

**DOI:** 10.1002/alz.091880

**Published:** 2025-01-09

**Authors:** Kylee Shivok, Elizabeth Affel, Robert C. Sergott

**Affiliations:** ^1^ The William H. Annesley, Jr, EyeBrain Center, Farber Neuroscience Institute at Thomas Jefferson University, Philadelphia, PA USA

## Abstract

**Background:**

FLIO a novel *in vivo* reproducible, non‐invasive imaging technology, measures fluorescence lifetime decay in two spectral channels for short‐lived retinal chromophores in two domains: Channel 1 emission wavelength 498‐560 nm corresponding to NADH and FAD/ATP function and Channel 2, 560‐720 nm wavelength corresponding to lipofuscin/lysosomal function. These data reflect the retinal mitochondrial molecular environment. Mitochondrial dysfunction has been recently explored as a cause of decreased synaptic function and cognitive decline in AD. Only very limited data with first generation FLIO technology were previously reported.

**Method:**

Twenty‐two individuals [48 eyes], 11 AD patients and 11 controls, underwent FLIO with second generation FLIO manufactured by Heidelberg Engineering. Imaging data were analyzed using SPCImage technology.

**Result:**

Patients with AD demonstrated a right shift towards longer wavelength chromophores for both channels 1 and 2. The mean fluorescence decay time of the eye was also extended, leading to much broader wavelength plateaus compared to relative small areas under normal parabolic curve found in controls. In this small cohort, no differences were observed between amnestic and non‐amnestic patients with AD. Mitochondrial changes were more pronounced with longer disease duration and severity.

**Conclusion:**

FLIO detected changes consistent with changes in the retinal mitochondrial molecular environment for both NADH, FAD, and lipofuscin not otherwise visible with other retinal imaging techniques. FLIO data add support to the hypothesis that mitochondrial dysfunction may contribute to AD neurodegeneration. FLIO may help to resolve the question of whether mitochondrial dysfunction is a primary event for AD and/or a secondary amplifier of the disease. Because it is non‐invasive, painless, reproducible, FLIO has promise as an imaging biomarker to aid in the diagnosis of AD as well as an outcome metric for longitudinal clinical trials.

